# Hyccin, the Molecule Mutated in the Leukodystrophy Hypomyelination and Congenital Cataract (HCC), Is a Neuronal Protein

**DOI:** 10.1371/journal.pone.0032180

**Published:** 2012-03-26

**Authors:** Elisabetta Gazzerro, Simona Baldassari, Caterina Giacomini, Veronica Musante, Floriana Fruscione, Veronica La Padula, Roberta Biancheri, Sonia Scarfì, Valeria Prada, Federica Sotgia, Ian D. Duncan, Federico Zara, Hauke B. Werner, Michael P. Lisanti, Lucilla Nobbio, Anna Corradi, Carlo Minetti

**Affiliations:** 1 Muscular and Neurodegenerative Disease Unit, G. Gaslini Institute, University of Genoa, Genoa, Italy; 2 Neuroscience Section, Department of Experimental Medicine, University of Genoa, Genoa, Italy; 3 Department of Neuroscience, G. Gaslini Institute, Genoa, Italy; 4 Biochemistry Section, Department of Experimental Medicine, University of Genoa, Genoa, Italy; 5 Department of Neurology and Genetics, San Martino Hospital, University of Genoa, Genoa, Italy; 6 Department of Cancer Biology, Kimmel Cancer Center, and the Stem Cell Biology and Regenerative Medicine Center, Thomas Jefferson University, Philadelphia, Pennsylvania, United States of America; 7 Department of Medical Sciences, School of Veterinary Medicine, University of Wisconsin, Madison, Wisconsin, United States of America; 8 Department of Neurogenetics, Max-Planck Institute of Experimental Medicine, Göttingen, Germany; University of Leicester, United Kingdom

## Abstract

“Hypomyelination and Congenital Cataract”, HCC (MIM #610532), is an autosomal recessive disorder characterized by congenital cataract and diffuse cerebral and peripheral hypomyelination. HCC is caused by deficiency of Hyccin, a protein whose biological role has not been clarified yet. Since the identification of the cell types expressing a protein of unknown function can contribute to define the physiological context in which the molecule is explicating its function, we analyzed the pattern of Hyccin expression in the central and peripheral nervous system (CNS and PNS). Using heterozygous mice expressing the b-galactosidase (LacZ) gene under control of the Hyccin gene regulatory elements, we show that the gene is primarily expressed in neuronal cells. Indeed, Hyccin-LacZ signal was identified in CA1 hippocampal pyramidal neurons, olfactory bulb, and cortical pyramidal neurons, while it did not colocalize with oligodendroglial or astrocytic markers. In the PNS, Hyccin was detectable only in axons isolated from newborn mice. In the brain, Hyccin transcript levels were higher in early postnatal development (postnatal days 2 and 10) and then declined in adult mice. In a model of active myelinogenesis, organotypic cultures of rat Schwann cells (SC)/Dorsal Root Ganglion (DRG) sensory neurons, Hyccin was detected along the neurites, while it was absent from SC. Intriguingly, the abundance of the molecule was upregulated at postnatal days 10 and 15, in the initial steps of myelinogenesis and then declined at 30 days when the process is complete. As Hyccin is primarily expressed in neurons and its mutation leads to hypomyelination in human patients, we suggest that the protein is involved in neuron-to-glia signalling to initiate or maintain myelination.

## Introduction

Hypomyelinating leukoencephalopathies of the central nervous system (CNS) are inherited white matter disorders (WMDs) characterized by permanent myelin deficiency. The term “hypomyelination” also applies to congenital disorders of the peripheral nervous system (PNS) characterized by hypomyelination in the presence or absence of signs of active demyelination [1]–[3].

The myelin sheath is a modified plasma membrane wrapped with a spiral pattern around axonal segments between the nodes of Ranvier. This highly specialized membrane is composed of multiple layers of myelin that have a protein-lipid-protein-lipid-protein architecture, and are modified extensions of oligodendrocytes in the CNS, or SC in the PNS. In both the CNS and PNS, the deposition and maintenance of myelin is complex and involves different cells and several axo-glial signalling pathways [4], [5] that have only partly been revealed [6].

Hypomyelination and Congenital Cataract, HCC (MIM #610532), is an autosomal recessive disorder first identified in five unrelated families with ten subjects affected by congenital cataract and diffuse cerebral and peripheral hypomyelination [7], [8]. While bilateral cataract was present at birth or within the first month of life, developmental delay was noticed at the end of the first year of life after initially normal psychomotor development. The neurological picture was characterized by pyramidal and cerebellar signs as well as muscle weakness and wasting of the lower limbs, indicating also PNS involvement. Indeed peripheral neuropathy was confirmed by neurophysiological and neuropathological studies. The clinical course was slowly progressive and the majority of patients became wheelchair-bound at around 8-9 years of life. Brain magnetic resonance imaging (MRI) showed diffuse hypomyelination with superimposed areas of abnormal signal intensity consistent with increased water content [9]. Sural nerve biopsies were characterized by a slight to severe reduction of myelinated fiber density with several axons surrounded by a thin myelin sheath or devoid of myelin. Uncompacted myelin sheaths, which in some fibers appeared redundant and irregularly folded, were occasionally seen [7].

HCC patients are affected by mutations in the gene *FAM126A* (previously named DRCTNNB1A). *FAM126A* encodes for a 521aa protein of unknown function, which we named Hyccin. In the first description of the disease, we identified two mutations affecting a splice-site, while the third one was a missense. At the protein level, all three mutants lead to absence or severe reduction of Hyccin protein [7].

Subsequently, the clinical spectrum of HCC was extended with the identification of a consanguineous family with a large intragenic deletion encompassing two exons of the *FAM126A* gene [10]. Notably, these patients did not display congenital cataract . Most recently Biancheri et al. (Archives of Neurology, in press) reported nine novel HCC patients from seven unrelated families. Although the latter study found significant clinical variability in the occurrence and age of onset of cataract as well as the severity and progression of neurological symptoms, MRI features of hypomyelination combined with increased periventricular white matter water content are consistently observed, distinguishing HCC from other forms of hypomyelinating leukoencephalopathies.

In order to define the cellular origin of HCC pathogenesis, we investigated Hyccin expression in the nervous system.

We found that Hyccin is predominantly expressed in the CNS, where it is localized in neurons but not in myelinating cells. In peripheral neurons, Hyccin is of low abundance and detectable only at early postnatal ages.

Moreover, we show that in DRG neurons and SC mixed cocultures, an *in vitro* model of myelination, the expression of Hyccin precedes the phases of active formation of the myelin sheath.

## Results

**Figure 1:**
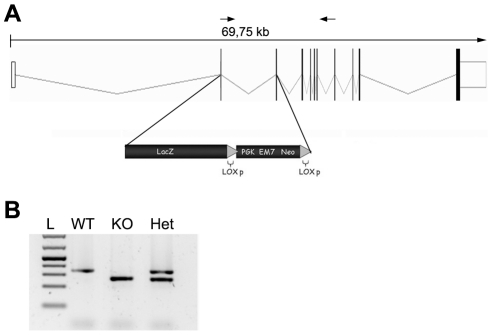
Generation of mice expressing lacZ under control of the Hyccin gene regulatory elements. Panel A: Schematic representation of the *Fam126a* locus and engineering of a *Fam126a* -LacZ allele. A segment containing exons 2 and 3 were replaced with a cassette containing a modified β-galactosidase coding sequence (LacZ) and a drug resistance minigene comprised of the phosphoglycerate kinase promoter (PGKp), a bacterial promoter (EM7p) and neomycin phosphotransferase (Neor), flanked by loxP sequences. The numbers below the line representing the genomic sequence indicate base pairs. Panel B: A representative PCR analysis of tail genomic DNA from wild-type (WT), heterozygous (Het) homozygous LacZ knockin mice and documenting *Fam126a* gene deletion.

To analyze the cellular expression of the Hyccin molecule, we took advantage of the promoterless LacZ gene (encoding a cytoplasmic b-galactosidase) integrated into the *Fam126a* locus of heterozygous (Het) Hyccin mice to express LacZ under control of the regulatory elements of the *Fam126a* gene. The endogenous *Fam126a* locus was targeted for modification by homologous recombination in embryonic stem (ES) cells using the selectable marker Neo. Fam126a coding sequence spans from exon 2 to exon 10 of the gene. The mutant allele was designed to replace the first coding exon (exon #2) (starting from amino acid 3), the intron in between and exon # 3 with a LacZ-Neo cassette (Fig. 1A–B). This deletion included the site of the IVS2+1G-A mutation, which was identified in a subgroup of our HCC patients and which leads to skipping of exon #2 with the generation of an aberrant Fam126a transcript and absence of Hyccin protein by immunoblot analysis [7].

The construct was delineated to drive LacZ under the control of the *Fam126a* promoter by removing the LacZ start codon and fusing it in frame with the first 3 amino acids of the *Fam126a* coding sequence. This design allowed us to detect the tissue location and developmental appearance of endogenous Hyccin by LacZ staining of Het mice. Hyccin Het mice were indistinguishable from wild type (WT) littermates based on body weight, cage behaviour, spontaneous locomotor activity, and reproductive performance (data not shown). The mutant allele is also disrupted regarding the expression of Hyccin and thus presents a functional null allele. The phenotypic consequences of homozygous deletion of Hyccin will be reported separately.

Hyccin-LacZ signal was evaluated in brain and sciatic nerves isolated from Het males at postnatal days P2, P10 and P30.

**Figure 2:**
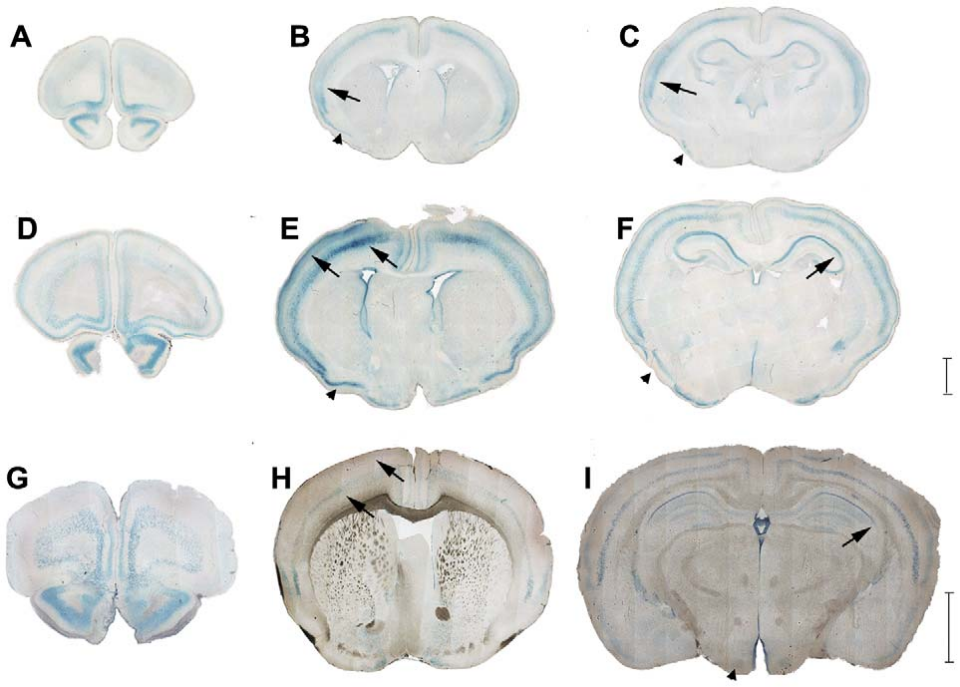
Analysis of β-galactosidase activity in the CNS from P2 (A,B,C), P10 (D,E,F), P30 (G,H,I) hyccin Het mice (n = 3). In the CNS, Hyccin is expressed in olfactory nuclei (A,D,G), in distinct layers of the neocortex (arrows in B,C, E,H) and in the hippocampal region with stronger expression in CA1 and CA2 fields as compared to CA3 (F,I arrows); dentate gyrus is negative. Other sites of expression include the prefrontal cortex, the piriform cortex (arrowheads B and D) and the ventromedial hypothalamic nuclei (I, arrowhead). Scale bars are 1000 micron (A,–F) and 2000 micron (G–I).

In the CNS, at P2 (Fig. 2 A, B and C) Hyccin gene activity was found in the olfactory nuclei and in the prefrontal cortex (A), in the piriform cortex (B and C arrowheads) and in the cerebral cortex with marked labeling in layer 5 (B and C arrows) and lower signal in layer 2. In addition, in the hippocampus LacZ labelling was detected in the pyramidal neurons of the CA1, CA2 and CA3 fields, while it was almost not detectable in the dentate gyrus. At P10 (Fig. 2 D,E and F), labeling was found in the same regions as at P2. However, in the cerebral cortex, expression in layer 2 was more prominent, with similar intensity than in layer 5 (E, arrows). In the hippocampus, labeling was more intense at the level of CA1 and CA2 fields compared to CA3 (F, arrow). At P30 (Fig. 2 G,H and I), the layers 2 and 5 of the cerebral cortex were positive at all levels along the anteroposterior axis with less intense signal compared to previous stages (H arrows). Interestingly, in the hippocampus the signal was confined to CA1 and CA2 fields, with a sharp boundary between CA2 and CA3 (I, arrow). Labeling was also present in olfactory nuclei (G), and in ventromedial hypothalamic nuclei (I, arrowhead).

**Figure 3:**
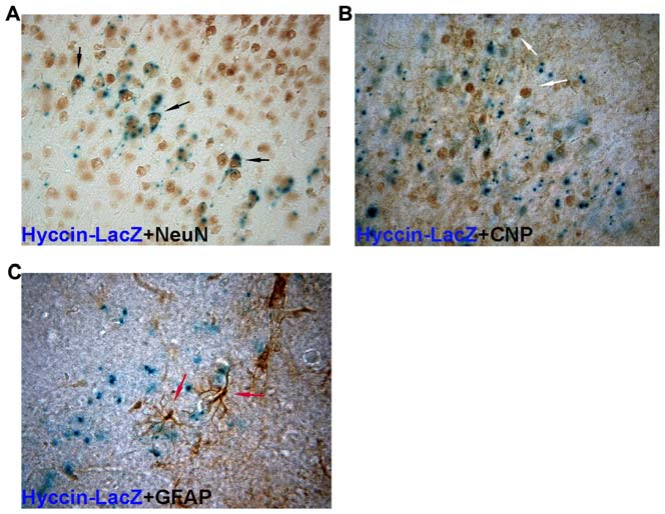
Immmunocolocalization analysis of Hyccin-LacZ activity and the markers NeuN (A), CNP (B) and GFAP (C) in the CNS hyccin from P10 Hyccin Het mice (n = 3). Cryostat sections (40 u thickness) from the CNS of LacZ stained Het mice were immunostained with primary antibodies raised against the different markers. Note that the LacZ signal colocalizes only with the NeuN nuclear neuronal signal. Representative pictures are shown.

To further confirm the principally neuronal expression pattern of the Hyccin gene, coronal sections of previously LacZ stained brains were probed with antibodies directed against the neuron-specific nuclear protein NeuN, the oligodendroglial 2′,3′-Cyclic-nucleotide 3′-phosphodiesterase (CNP) or the astrocyte marker glial fibrillar acidic protein (GFAP) (Fig. 3).

Notably, the LacZ signal was detected in cells immunopositive for NeuN (Fig. 3A), while it did not associate with CNP (Fig. 3B) or GFAP (Fig. 3C) markers.

To confirm the results independent of the gene targeting, we applied *in situ* hybridization and immunofluorescence to WT mice.

**Figure 4:**
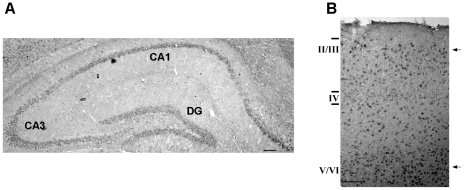
RNA in situ hybridization on brain sections from P30 WT mice reveals an expression pattern in agreement with principal neuronal expression. Coronal brain sections were hybridized with a Hyccin antisense 700 bp probe. Panel A: High-magnification image of hippocampus shows the strongest labeling of Hyccin in the CA1, 2 region compared to the CA3 region and dentate gyrus (DG). Scale bar is 200 µ. Panel B: High-magnification image of the cerebral cortex revealed a stronger labeling of Hyccin in deep cortical layers (V/VI) compared to superficial layers (II–III). Scale bar is 200 µ.

RNA *in situ*-hybridization was performed on coronal sections from P30 WT mouse brain via an anti-digoxygenin Hyccin 700 bp antisense oligonucleotide probe [11]. Hyccin signal was most intense at the level of the cortex and the hippocampus (Figure 4A and B). In the cerebral cortex, Hyccin labelling was expressed strongest in layer 5.

**Figure 5:**
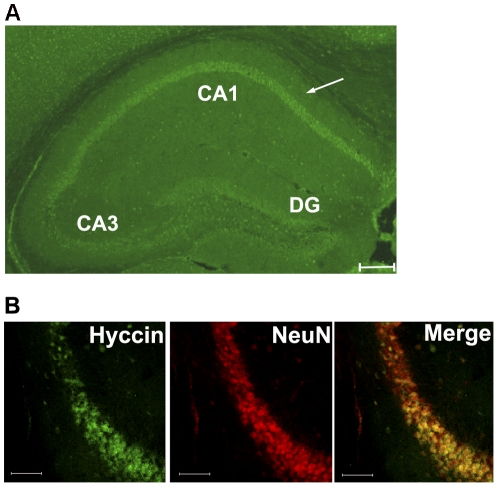
Immunofluorescence analysis of Hyccin on brain sections from P30 WT mice. Panel A: Coronal brain sections were probed with anti-hyccin polyclonal antibody. High-magnification image of hippocampus shows the most intense labeling of Hyccin in the CA1, 2 regions compared to the CA3 and dentate gyrus (DG). Scale bar is 200 µ. Panel B: Coronal brain sections were probed with anti-Hyccin polyclonal antibody or with anti-NeuN antibody. Hyccin and NeuN immunoreactivity were detected by respectively Alexa 499 and 546 conjugated secondary antibodies. High magnification image of CA1 region of the hippocampus. Note the colocalization between hyccin and the neuronal marker in the merge. Scale bars are 80 µ.

By immunofluorescence, Hyccin fluorescent signal was detected in the hippocampal segments where the labeling was strikingly more intense in the CA1 and CA2 regions when compared to the CA3 or to the dentate gyrus (Fig. 5A), in agreement with the gene expression pattern found by LacZ staining. Moreover, in consistency with the LacZ staining analyses, Hyccin colocalized with the NeuN marker (Fig. 5B). The specificity of the antiserum directed against Hyccin was determined by comparison of protein lysates from human healthy controls (n = 3) and HCC patient fibroblasts (n = 3) (mouse and human hyccin protein sequence are 97% homologous). Western blot analysis indicated that the antibody recognized a protein of the expected size of 54 kDa in extracts of control fibroblasts, while no signal was observed in patients' fibroblasts (Figure S1-A). These results were confirmed in HeLa cells, known to express hyccin protein, by peptide competition, in which the primary antibody directed against Hyccin was pre-incubated with a 5-fold molar excess of the immunizing peptide before being exposed to the antigen (Figure S1-B).

**Figure 6:**
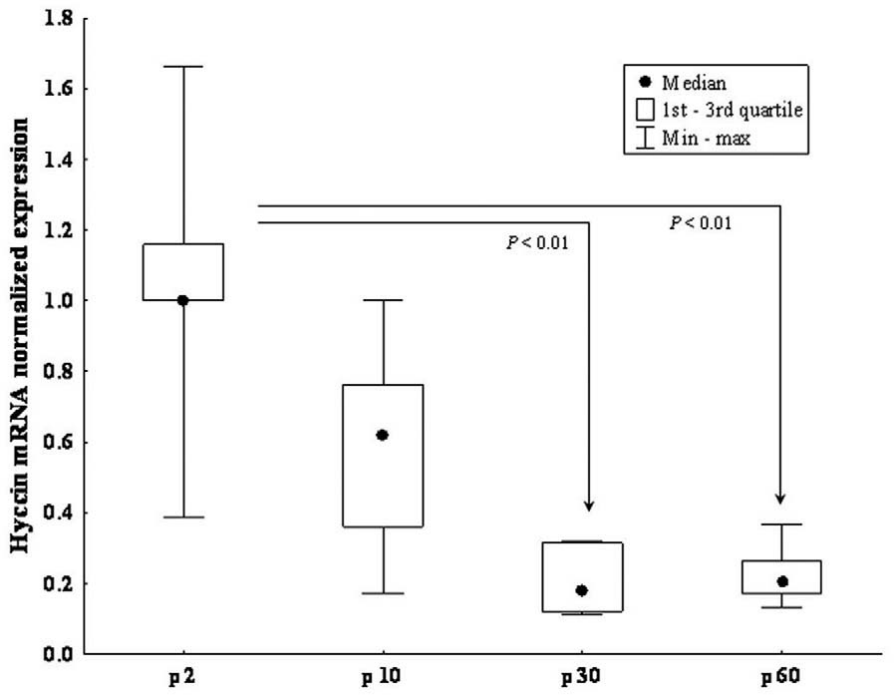
Real-time PCR analysis of Hyccin transcripts in total brain lysates from P2 (n = 12), P10 (n = 12), P30 (n = 6) and P60 (n = 6) WT mice. Results are expressed as mRNA fold increase relative to calibrator (p2) normalized to housekeeping genes (b-actin and GAPDH). Quantitative data were reported as median values with first and third quartiles (1st-3rd q) and minimum and maximum value (Min-max). * = p<0.01.

The temporal regulation of Hyccin expression in the CNS was quantified in total brain lysates isolated from P2, P10, P30, P60 WT animals by real-time PCR. Hyccin transcripts were significantly more abundant at postnatal days 2 and 10 and then decreased by approximately 75% in adult animals (Fig. 6).

As concerns the PNS, sciatic nerves from P2, P10, P30 and P60 Hyccin Het mice were stained with 5-bromoindolyl-o-galactopyranoside (Bluo-Gal) staining solution for optic and electron microscopy. After -galactosidic cleavage, Bluo-Gal precipitates in form of fine birefringent crystals, whereas X-gal gives rise to an amorphous precipitate. This property of Bluo-Gal results in greatly enhanced sensitivity of the staining method for LacZ and allows for optimal morphological resolution in electron microscopy [12].

At all ages examined, Bluo-Gal staining was not detected in peripheral nerves of Het mice by light or electron microscopy, indicating that b-galactosidase was not very abundantly expressed under control of regulatory elements of the Hyccin gene. Heterozygous *Ebf* 2 null mice, in which the *Ebf2* coding region was replaced by LacZ, were used as positive controls [13].

Interestingly, Bluo-Gal precipitate was detected in peripheral nerves of mice homozygously expressing LacZ under control of the Hyccin gene, though confined to postnatal day 2.

**Figure 7:**
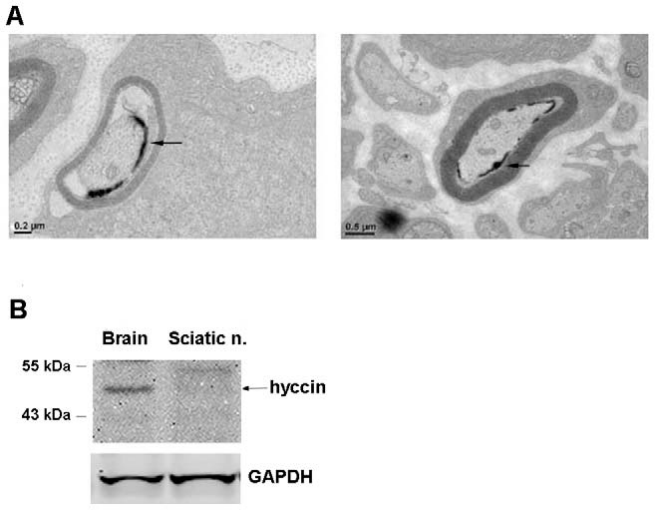
Analysis of Hyccin expression in the PNS. Panel A: Semithin sections of sciatic nerves isolated from Hyccin KO mice (n = 3) were stained with Bluo-Gal and analyzed by electron microscopy. Arrows point to Hyccin-LacZ signal. Panel B. Immunoblot analysis of hyccin protein in sciatic nerve lysates isolated from P10 WT mice as compared to total brain lysates. Note that Hyccin protein levels is very low in the mature sciatic nerve sample. GAPDH was utilized as a sample loading control.

In this cohort of mice, LacZ signal localized to axons surrounded by myelin-forming SC (arrowhead in Fig. 7A).

Hence, Hyccin protein levels were compared in total brain and sciatic nerve lysates by immunoblot analysis at the age of postnatal day P10.

Consistent with the Bluo-Gal staining, Hyccin protein levels were of very low abundance in sciatic nerves when compared to total brain lysates (Fig. 7B).

**Figure 8:**
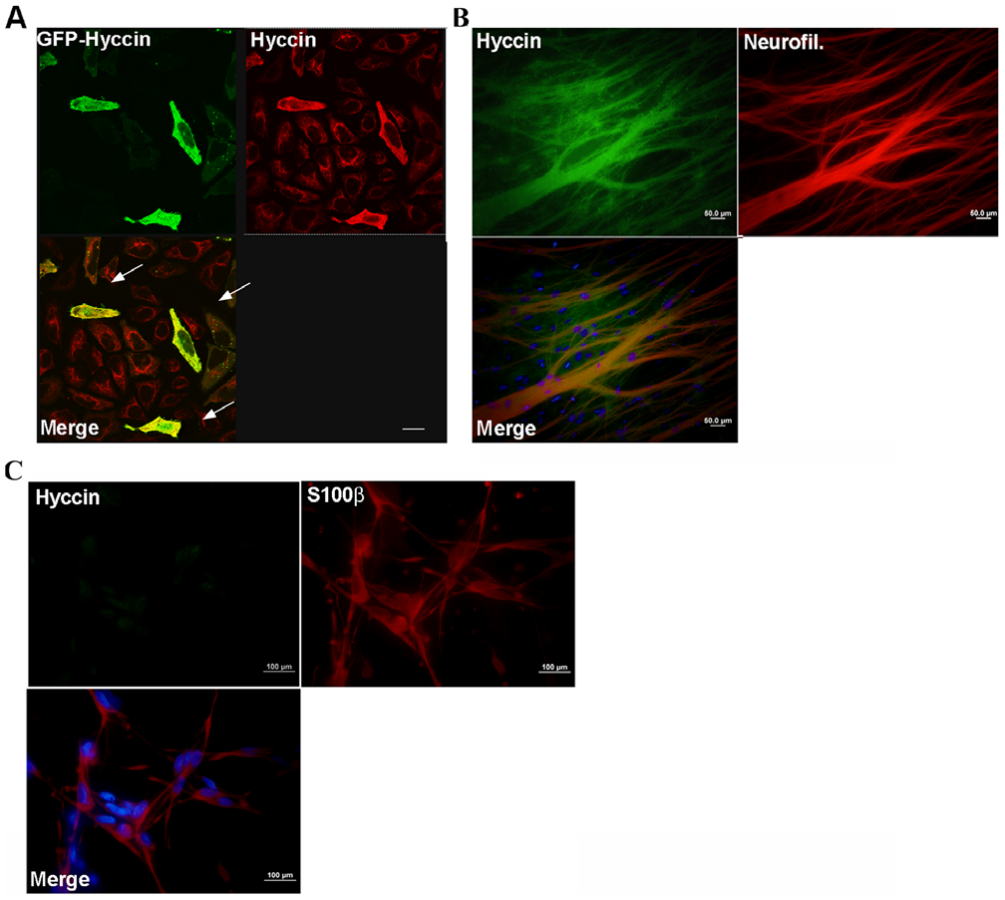
Immunofluorescence analysis of hyccin expression in DRG neurons and Schwann cell cultures. Panel A: Immunocolocalization of GFP-Hyccin transiently transfected in Hela cells and the anti-hyccin polyclonal antibody. Representative images of Hela cells transfected with GFP-Hyccin and immunostained with anti-hyccin polyclonal antibody 24 hr after transfection. Hyccin immunoreactivity was detected by Alexa546 conjugated secondary antibodies. The arrows in the merge panel show colocalization of the two signals in the cytosol. Final magnification 63×. Scale bars represent 100 µ. Panel B: DRG neurons cultures were immunostained with an anti-hyccin antibody and an anti-phosphorylated neurofilament. Hyccin and Neurofilament immunoreactivities were detected respectively by Alexa488 and Alexa546 conjugated secondary antibodies. The merge panel shows positivity of the two signals in the neurites. Final magnification 40×. Panel C: Schwann cells cultures were immunostained with an anti-hyccin polyclonal antibody and an anti-S100 monoclonal antibody. Hyccin and S100 immunoreactivities were detected respectively by Alexa488 and Alexa546 conjugated secondary antibodies. Final magnification 100×.

To further define the expression of Hyccin in the PNS, primary rat SC and DRG sensory neurons were immunostained with the anti-Hyccin antibody. The specificity of the antibody for immunocytochemistry was confirmed by colocalization studies in HeLa cells transiently transfected with a GFP-hyccin expression vector (Fig. 8A).

Hyccin positivity was observed along the neurites, while no specific signal was detected in SC. These results were confirmed by double labelling of Hyccin and respectively phosphorylated neurofilament and S100 protein. (Fig. 8 B,C). Anyhow, further experiments are needed to clarify the expression and function of Hyccin in the PNS.

**Figure 9:**
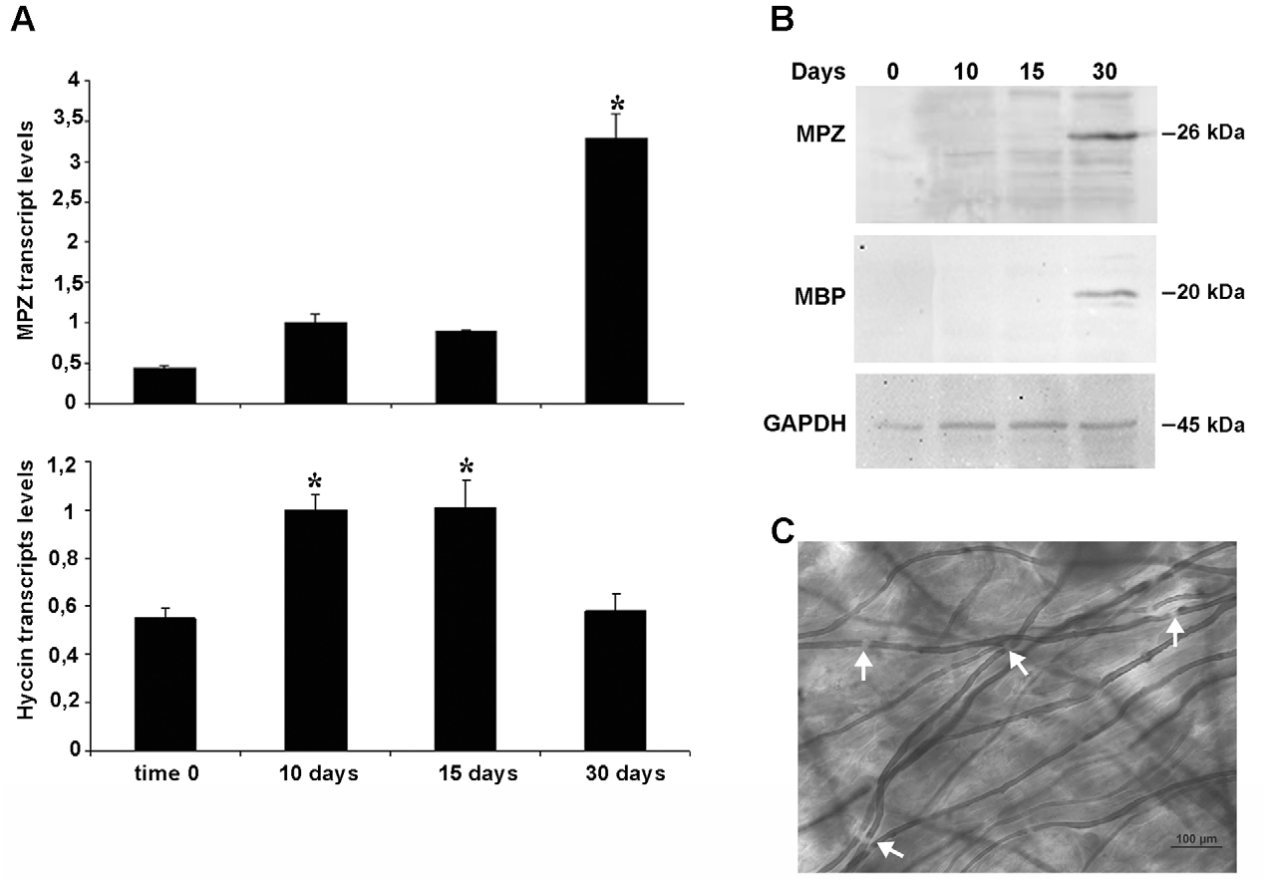
Hyccin expression levels in organotypic DRG cultures. Panel A: Cells were maintained in culture for the times indicated and analysis of MPZ mRNA was utilized as a reference for myelination in the culture. Results are expressed as mRNA fold increase relative to calibrator (10 days) normalized to housekeeping genes (b-actin and GAPDH) For each sample data are represented as means ±SEM (n = 3) and .p values were <0.05 compared to control. Panel B: Total cellular lysates were isolated at the times indicated and subjected to immunoblot analysis for the myelin markers MPZ and MBP. An antibody against GAPDH was used as an internal control. Panel C: At 30 days cell cultures were fixed, stained with 1% Sudan Black in 70% ethanol and analyzed by optic microscopy. Bright-field micrographs confirm the presence of myelin sheaths with Ranvier nodes (white arrows) surrounding th axons.

Finally, to correlate the temporal evolution of Hyccin expression with active myelinogenesis, we established a 30 days organotypic DRG culture. The transcripts of myelin protein zero (MPZ) were quantified to control the correctness of the process of myelination. Interestingly, Hyccin mRNA levels increased during the first 10–15 days of the culture, when myelin forming cells are differentiating, and then decreased of about the 40% at 30 days when SC fully expressed the myelin markers MPZ and myelin basic protein (MBP) and when myelin sheath were formed (Fig. 9, ABC).

In conclusion, in the CNS, Hyccin is expressed in a subset of the neurons, and its transcripts are significantly more abundant in young mice at early stages of postnatal development correlating with the onset of myelination. The abundance of Hyccin is lower in the mature PNS, where the protein was detected only in null Hyccin mice carrying two copies of the LacZ allele.

Finally, in an *in vitro* model of myelinogenesis, i.e. a mixed population of SC and sensory neurons, Hyccin is up-regulated in the steps of active myelination thus supporting the hypothesis of its active involvement in the formation of the myelin sheath.

## Discussion

Our study aimed to establish the cellular expression in the nervous system of a novel protein, Hyccin, responsible for the etiology of the autosomal recessive hypomyelinating disorder HCC.

In the past few years our understanding of the nosology and physiopathology of inherited white matter diseases has greatly improved, though a significant number of patients with cerebral white matter involvement still remain without diagnosis despite extensive investigations. Hypomyelination is the single largest category among undiagnosed cases [3], [14]–[17]. The prototype of defined hypomyelinating leukoencephalopathies is Pelizaeus-Merzbacher Disease (PMD) caused by a primary defect of myelin proteolipid protein (PLP) expressed in oligodendrocytes; however, other hypomyelinating disorders are related to primary neuronal or astrocytic dysfunctions, including Salla disease caused by SLC17A5 gene mutations, Pelizaeus Merzbacher-Like Disease due to GJA12 mutations, or Alexander disease caused by mutations affecting the astrocytic GFAP [18]–[24]

The discovery that the hereditary leukoencephalopathy HCC is caused by mutations affecting the gene *FAM126A*, which encodes for Hyccin, a protein of unknown function, raised the question how this molecule may function, and why functional loss may lead to hypomyelination.

Hyccin is expressed at low levels in a variety of adult tissue, and its transcripts are most abundant in the heart, spleen, and cerebral cortex (data not shown).

Phylogenetic and multiple alignment analysis of human *FAM126A* identify a homolog called *FAM126B*, whose function and pattern of expression are unknown. Phylogenetic analysis and software-based prediction tools do not allow any classification of Hyccin into any specific protein family, and do not suggest any previously defined functional domain.

Therefore, as a step towards understanding the pathogenesis of HCC, we established the specific expression pattern of Hyccin in the CNS and PNS. For this purpose, we generated a new mouse model expressing LacZ under control of the regulatory elements of the Hyccin gene and thus allow monitoring Hyccin gene activity by b-galactosidase staining. LacZ activity was evaluated in early postnatal mice (P2 and P10) and adult animals. Notably, the protein is differentially expressed in neuronal cells in distinct layers of the cerebral cortex and in the hippocampal region. Importantly Hyccin was not detected in cells which immunostained positive for the oligodendroglial marker, CNP, or the astrocyte molecule GFAP.

Since LacZ signal reflects activity of the modified allele but is only an indication for the expression of Hyccin (as post-transcriptional effects may have an influence as well), these results were confirmed independently by RNA *in situ*-hybridization and immunofluorescence analysis. Confocal microscopy of Hyccin signal allowed also a quantitative comparison of its labeling in the different regions of the hippocampus.

The detection of strong expression of a gene involved in myelination in the CA1 and CA2 regions of the hippocampus, when compared to CA3 or the dentate gyrus is not surprising. Indeed, these regions are characterized by densely packed pyramidal somata which result immunopositive for Hyccin and NeuN signal and which are characterized by fully myelinated long-projecting axons. In the CA3 region the pyramidal neurons display a minor density, while in the dentate gyrus the neuronal population is composed of interneurons and granule cells, which only project to CA3 through non-myelinated mossy fibres.

Myelination is a strictly regulated process. In the mouse, it starts approximately at birth in the spinal cord, and in the brain is largely accomplished in almost all regions by around 45–60 postnatal days. When tested by real-time PCR on total brain lysates, Hyccin expression is highest in newborn mice and at early stages of postnatal development and then decades in adult animals, thus indicating an up-regulation of the molecule expression correlating with an early phase of myelinogenesis.

In the PNS, Hyccin-LacZ signal was not detected in Het animals at any age examined, and was identified only by electron microscopy at postnatal day 2 in homozygous mice in which both alleles are expressing LacZ.

When tested *in vitro*, in primary cultures of SC or DRG neurons, Hyccin was visualized on the neurites, while it was absent in SC. Notably, temporal analysis of Hyccin expression in organotypic cultures of DRG neurons and SC highlighted its possible role in myelination. This *in vitro* system has been extensively used to study development and regeneration of peripheral nerves, and diseases including hereditary sensory and motor neuropathies, and it constitutes a valuable model to analyze particularly the early steps of myelination [25]–[30]. In this model, Hyccin was up-regulated at days 10 and 15, a step of the culture which precedes myelin formation and the expression of classical myelin markers. This result seems to be consistent with the hypothesis of Hyccin as an axonal signal instructing myelination and stimulating SC to synthesize myelin proteins and lipids, and to extend myelin sheaths.

Previous reports from the literature showed Hyccin transcripts in neurons as well as in cells of the oligodendrocytic and astrocytic lineage [31], [32]. It is well possible that indeed a minimal amount of Hyccin mRNA may be expressed also in cells other that neurons but that the protein is not detectable or not present.

In our paper we analyzed mainly Hyccin protein pattern of expression and to support the data we followed an integrated approach based on *in vivo* b-galactosidase gene activity data plus immune-based experiments.

Moreover, Hyccin main neuronal expression here shown is in accordance with brain and spinal cord data reported on the Allen Brain Atlas website, a database of genes expression pattern of the mouse brain completed by RNA in situ hybridization (http://www.brain-map.org).

In this work, we have shown that Hyccin is neuronally expressed. Considering the hypomyelination in patients with mutations affecting the Hyccin gene, we suggest that this protein is part of the signals that regulate neuron-to-glia cross-talk at the onset of myelination, or regarding myelin maintenance. Indeed, neurons utilize a battery of signals to control the differentiation of associated myelinating glial cells. In the PNS, axonal molecules involved in the induction or maintenance of myelin include Neuregulin-1 [33], [34] the shedding enzymes BACE and TACE [35], [36], BDNF signaling to the neurotrophin receptor p75 [37], and axonal prion protein [38], while axonal signalling to myelinating glia has been less well understood in the CNS [6]. Hyccin might be a co-factor or regulator of neuronal molecules already known to mediate the neuron-glia interplay; however its relevance for CNS myelin indicates that the protein may be an important part of an independent, yet to be identified signalling mechanism.

## Materials and Methods

### Antibodies

The antibodies and dilutions used in the study include the following: polyclonal antibody against Hyccin (D-15) (Santa Cruz; Biotechnology) (1∶50 for tissue immunofluorescence [IF], 1∶500 for Western blot [WB]); polyclonaly anti-hyccin antibody (Novus Biologicals) (1∶100 for immunocytochemistry); monoclonal antibody against phosphorylated (SMI31) neurofilaments (NF) (Sternberger Monoclonals Inc.) (1∶250 for IF); monoclonal anti-Glial Fibrillary Acidic Protein (GFAP), clone GA5 (Millipore) (1∶400 for immunohistochemistry [IHC]; monoclonal anti-neuronal Nuclei (NeuN), clone A60 (Millipore), (1∶1000 for IHC and 1∶150 in IF); monoclonal anti- 2′,3′-cyclic nucleotide 3′ phosphodiesterase (CNP), clone 11-5B (Millipore), (1∶50 for IHC); Fab fragments anti-Digoxigenin-Alkaline phosphatase (AP), (1∶2000 for *in situ*-hybridization), (Roche); monoclonal anti-myelin basic protein (MBP) clone MAB382, (Chemicon) (1∶3000 in WB); monoclonal anti myelin protein zero () (1∶1000 in WB); polyclonal anti-glyceraldeyde 3 phosphate deydrogenase (I-19) (Santa Cruz, Biotechnology), a monoclonal anti-S100 antibody (Abcam), a monoclonal anti-myelin protein zero (MPZ), P07 extracellular domain, Astexx Ltd. & Co. KEG, Graz, Austria. Rabbit anti-goat IgG horseradish peroxidase-conjugated (1∶4000 for WB) was from ZYMED (Invitrogen), while the anti-mouse Dako EnVision+ System-HRP Labelled Polymer was from Dako Group. Hyccin fluorescent signal was detected following tyramide signal amplification according to manufacturer's instructions (PerkinElmer Life Sciences).

In confocal microscopy, secondary antibody reaction was carried out using donkey anti mouse Alexa Fluor 488, donkey anti goat Alexa Fluor 633, rabbit anti mouse Alexa Fluor 633, chicken anti rabbit Alexa Fluor 657 antibodies (1∶600) (all from Molecular Probes Inc.).

### Generation and genotyping of Hyccin knock-out mice

The mouse *Fam126a* locus spans 130 kilobases (kb), and exons 2 to 11 encode the protein. VelociGene technology was used to replace a segment of *Fam126a* including exons 2 and 3 corresponding to an 11-kb genomic region (base pairs [bp] 2378 to 2473 of exon 2, 2473 to 13230 of intron 2 and 13231 to 13324 of exon 4 [Ensemble Accession Number: ENSMUSG00000028995; GenBank Accession number: NM_053090] with a β-galactosidase (LacZ)/neomycin (Neor) cassette [39]. The replacement cassette was formed by the LacZ reporter gene and by a phosphoglycerate kinase promoter-Neor selection cassette flanked by LoxP sequences. Briefly, a bacterial artificial chromosome (BAC) containing *Fam126a* coding region and 800 bp of total flanking sequences (clone X from release II of a 129/SvJ BAC library obtained from Incyte Genomics; Wilmington, DE) was modified to generate a BAC-based targeting vector, which was linearized and used to replace *Fam126a* gene in F1H4 (C57BL/6∶129 hybrid) mouse embryonic stem (ES) cells. Correctly targeted ES cells were identified by using the loss of native allele (LONA) assay, as described [11]. Two independently correctly targeted ES cell lines were used to generate chimeric male mice that were complete transmitters of ES-derived sperm. Chimeras were bred to C57BL/6 females to generate F1 heterozygous mice, which were genotyped by LONA assays and identified by LacZ histochemical assays. F1 mice were crossed with C57BL/6 mice and the F2 offspring was used to maintain the line in a mixed 129/C57BL/6 genetic background. For each experiment, Hyccin heterozygous (Het) male mice derived from F2 heterozygous intercrosses were used.

Genotyping was completed by PCR on genomic DNA isolated from tail samples.

The sequences of primers used for the LacZ and for *Fam126a* genes are the following:

LAC Z allele (360 bp): Forward: 5′ AAA GAC GCA GTT CTG CAT GG 3′, Reverse: 5′ TCA TTC TCA GTA TTG TTT TGC C 3′ FAM126a WT allele (280 bp): Forward:5′ GTA CCA AAC ACC AGC ATG GA 3′, Reverse: 5′ TCA CTC TGT GGC TCC TGG AT 3′.

All the Animal experiments were approved by the Animal Care and Use Committee of Istituto di Fisiologia Clinica, CNR, Pisa, Italy (Project # 187).

### Cell cultures

HeLa cells were maintained in Dulbecco's modified Eagle's medium (DMEM), supplemented with 10% fetal calf serum (FCS), penicillin and streptomycin in a humified 5% CO_2_ atmosphere at 37°C. All cell culture products were purchased from Gibco/Invitrogen (Merelbeke, Belgium).

The GFP-tagged ORF clone of *FAM126A* (GFP-hyccin) purchased from Origene was transiently transfected in Hela cells using the Turbofect ™ in vitro Transfection Reagent (Fermentas) according to manufacturer's instructions (1 ug DNA complexed with 1,5 u/well of transfection reagent). Hyccin expression was evaluated by immunofluorescence or western blot analysis 24 h after transfection.

Organotypic dorsal root ganglia (DRG) cultures were established from 15-day-old embryos using previously established methods [12]. After extracting them using a sterile technique, 30 to 35 DRG were removed from each embryo, pre-treated with trypsin (Hanks'solution 0.25%), and triturated to provide a suspension of DRG cells in a medium supplemented with 15% bovine calf serum, and nerve growth factor 5 ng/mL final dilution. This suspension was plated on a collagen substrate in flexible molded plastic Aclar dishes, and 15×10^4^ cells were placed in each dish. As for each embryo we established 4 cultures, we obtained enough material to perform a time-course (30 days) analysis of hyccin expression and myelination features by both molecular biology and light microscopy evaluations.

For myelination staining, DRG cultures were washed in PBS, fixed in Trump's fixative at 4°C overnight, post-fixed the following day in 2% osmium tetroxide and stained with 1% Sudan Black in 70% ethanol. Each culture was assessed for the presence or absence of myelin and for the morphological appearance of all observed fibres.

Establishment of primary SC and DRG neurons cultures has been described previously [29], [30].

Neuronal cultures were established from 15 day-old wild-type rat embryos. After extracting them using a sterile technique, 35 to 40 DRG were removed from each embryo, pre-treated with trypsin (Hanks'solution 0.25%), and triturated to provide a suspension of DRG cells in a medium supplemented with 15% newborn calf serum (NCS), ascorbic acid (100 ug/mL final dilution) and nerve growth factor (NGF) (5 ng/mL final dilution). This suspension was plated on collagen-coated ACLAR dishes at a density of 15×10^4^ cells/dish. After 48 hr, the cells were treated with Fudr medium (neurobasal medium, 15% NCS, NGF, ascorbic acid and 10^−5^ M 5-fluoro-2′-deoxyuridine and uridine) (Invitrogen, Srl, Italy) for two 48-hr cycles to eliminate fibroblasts growth. Cultures were used for molecular and immunocytochemistry evaluations.

SC cultures were established from 3 day-old rat pups. Sciatic nerves of the newborn rats were dissected and dissociated [15]. SC were grown in DMEM/F12 medium (Invitrogen) supplemented with 10% fetal calf serum (FCS), penicillin and streptomycin for 48 hr. Cytosine arabinoside (Ara-C) 10^−5^ M was added after 48 hr and the treatment prolonged for further 48 hr resulting in SC cultures that were 99% pure and were used for molecular and immunocytochemistry evaluations.

Primary fibroblasts were isolated from cutaneous biopsies of HCC patients (n = 3) and healthy controls (n = 3). Written informed consent was received from all participants.

### Quantitative PCR

Quantitative real-time PCR experiments (qRT-PCR) were carried out on total brain lysates from C57/BL6J WT mice (P2, P10, P30, P60), different organs from C57/BL6J WT mice (P30) and from rat Dorsal Root Ganglion (DRG)-Schwann cocultures (10 days, 15 days, 30 days of culture).

For DRG, and Schwann cells total RNA was extracted using the RNeasy Micro Kit (Qiagen) according to the manufacturer's protocol including DNase treatment; for mouse tissues total RNA was extracted using the RNeasy Lipid Kit (Qiagen). Quality and quantity of RNA were analyzed using a NanoDrop spectrophotometer. The cDNA was synthesized from 100 ng of total RNA with the iScript cDNA Synthesis Kit (Bio-Rad Laboratories). Each RNA sample was controlled for genomic DNA contamination without reverse transcriptase addition into cDNA synthesis mixture. qRT-PCR was performed in triplicate with the 2× Power Sybr Green PCR Master Mix (Applied Biosystem) in CFX96 Real-time PCR detection system (Bio-rad). The 15 µl PCR mixture contained diluted cDNA corresponding to 3 ng of total RNA and 0.2 µM of each primer. Relative expression levels were normalized to calibrator sample by using the comparative Ct (ΔΔCt) method and the geometric average of a set of two housekeeping genes [40]–[42]. (GAPDH, b-Actin) by the Bio-Rad CFX manager software. For each specific primers set, the efficiency was >95% and a single product was seen on the melting curve analysis. Specific primers for FAM126a (NM_001191969 rattus norvegicus, NM_053090 mus musculus), beta-actin (ACTB, NM_031144 rattus norvegicus, NM_007393 mus musculus), Glyceraldehyde-3-phosphate dehydrogenise (GAPDH, NM_017008 rattus norvegicus, NM_008084 mus musculus) and Myelin Protein Zero (MPZ, NM_017027) were designed through Beacon Designer 2.0 Software and are listed in Methods S1.

### RNA *In situ*-hybridization of mouse brain

*In-situ* hybridization was performed on brains isolated from P30 C57/BL6J wild-type (WT) mice. The mice were anaesthetized with isofluorane (Isofluorane-vet, Merial) and decapitated. Upon sacrifice, brains were incubated in PAF 4% in PBS ON at 4°C, cryo-protected with sequential incubations with 15% sucrose/PBS O.N. at 4°C and 30% sucrose/PBS until submerged.

Then brains were embedded in OCT and immediately frozen in cold isopentane. Sections (10 um) were cut by a cryostat and mounted on glass slides (VWR International).

Sections were delimitated with DakoPen (Dako Group) washed in PBS permeabilized with RIPA buffer two times for 10 min and fixed in PAF 4% in PBS for 10 min at RT.

After three washes in PBS, sections were treated with an acetylation step with 0.25% thrietanolamine and acetic anhydride.

The pre-hybridization reaction was performed for 1 h at 70°C using the hybridization buffer containing 50% formamide/2× NaCl 150 mM, Na_3_Citrate 15 mM (SSC), 5× Denhardt Solution, Herring sperm DNA 500 µg/ml (Invitrogen), Yeast RNA 250 ug/ml (Ambion).

We used a set of digoxigenin-labeled riboprobes that span 700 bp of *FAM 126a* transcript.

Probes were obtained from PCR amplification (primers probe 1: mDRC sense 1: T7 TAATACGACTCACTATAGGAGATACAGAATTAACAGGTC; mDRC antisense1: SP6 ATTTAGGTGACACTATAGATGCAGACAGAGTGACGCT; probes 2: mDRC sense 2: T7 TAATACGACTCACTATAGGGAAAGACAAGAGTTCTTTAG; mDRC antisense 2: SP6 ATTTAGGTGACACTATAGAAATGTCAATACTTCAAACCT).

PCR products (20 µg) were purified with QIAquick PCR Purification Kit (QIAGEN) and used to generate the sense and antisense riboprobes (Riboprobe Combination system-SP6/T7, Promega Corporation, DIG RNA Labeling Mix, Roche Diagnostic). Probes were purified by RNA Cleanup and Concentration Kit (QIAGEN) and measured with NanoDrop spectrophotometer. Each riboprobe was transcribed as sense, which specifically labeled the target mRNA, and antisense, to control for unspecific binding or for background staining due to the endogenous alkaline phosphatase activity of the tissue.

For the hybridization reaction, slides were incubated in hybridization buffer with addition of 100 ng/ml of probes ON in a humidified chamber at 70°C. The washing steps included incubations with formamide 50%/2× SSC, 0.1% Tween-20 (post-hybridization buffer) for two times 1 h at 70°C, Subsequently the samples were treated with Buffer B1 (Maleic acid 100 mM pH 7.5, NaCl 150 mM, 0.1%Tween-20) blocked with 10% Fetal Calf Serum (FCS)/B1 for 30 min at RT and incubated ON at 4°C with anti-digoxigenin-AP antibody in 10%FCS/B1.

Slides were washed twice with B1, incubated in Tris-HCl 100 mM pH 9.5, MgCl_2_ 50 mM, NaCl 100 mM, 0.1% Tween-20 for 30 min at RT, and finally detected with Nitroblue tetrazolium Chloride (NBT)- 5-Bromo-4-Chloro-3-indolyl phosphate, toluidine salt (BCIP).

When the colour reaction was completed, sections were washed with 0.1%Tween-20/PBS and dried [11].

### Expression analysis of the β-galactosidase reporter gene and immunohistochemistry (IHC)

Brains were isolated from postnatal day (P) P2, P10, P30, P60 Hyccin Het mice , fixed 3 h with fixing solution (glutaraldehyde 0.2%, ethylene glycol tetracetic acid (EGTA) 100 mM, 2% Paraformaldehyde (PAF) in PB buffer, i.e. phosphate buffer 0.12 M, pH 7.4), washed once with PB buffer and three times with Wash Buffer (MgCl_2_ 0.1 M, 1%, sodium deoxycholate 2% NP-40 in PB buffer).

The tissues were embedded in 2.5% agarose in PB buffer. Coronal sections (100 um) were cut with a microtome HM 650 V (Bioptica), washed 3 times with Wash Buffer and with PB, and then incubated with X-Gal solution (X-Gal 100 mg/ml, ferrocyanide potassium 2 mg/ml, ferricyanide potassium 1.64 mg/ml in Wash Buffer) for 3 h (P2, P10 mice) and ON (P30, P60 mice) at 37°C.

Sections were analyzed with Neurolucida software (MicroBrightField) connected to a Nikon E-800 microscope via a colour CCD camera.

For the colocalization studies, coronal sections from brains of P30 Hyccin Het mice were cut with the microtome (1500 um) and processed for X-Gal staining. Following the staining and the washes, tissues were incubated in 4% PAF/PBS for 1 h at 4°C, cryo-protected with incubation in 30% sucrose/PBS ON at 4°C, embedded in OCT (Tissue-Tek Sakura Finetek) and stored at −80°C.

Free-floating coronal sections (40 um) were cut on a cryostat (Leica CM1900UV), processed with 0.5% SDS/PBS for antigen retrieval, incubated with 0.3%, H_2_O_2_ 1,0% CH_3_OH/PBS, washed three times in PBS, blocked with 15%/Horse Serum/0.1% Tween/PBS (blocking solution) for 30 min at RT and incubated with the primary antibodies diluted in blocking solution ON at 4°C. The following day the sections were washed three times with PBS, blocked for 30 min in blocking solution and incubated with the secondary antibody anti-mouse Dako EnVision for 30 min a RT in the dark. Sections were washed with PBS, were developed with DAB (Dako Group), and mounted with Vectashield Mounting Medium (Vector Laboratories).

### Immunofluorescence analyses

Brains were isolated from P30 C57/BL6J WT mice and fixed in 4% PAF/PBS ON at 4°C and cryo-protected with sequential incubations with 15% sucrose/PBS O.N. at 4°C and 30% sucrose/PBS until submerged. The tissues were hence incubated in a 1∶1 solution containing OCT and 30% sucrose/PBS for 1 hour in agitation at RT, and finally embedded in OCT.

Free-Floating sections (40 um) were cut on a cryostat, were rinsed with PBS, incubated with 0.5 M Ammonium Chloride for antigen retrieval, rinsed with PBS, blocked with 3% Bovine Serum Albumin (BSA)/0.1% TritonX-100 (blocking solution) in PBS for 30 min at RT, and incubated with primary antibodies diluted in blocking solution ON at 4°C.

Sections were washed three times with 0.1% Triton X-100/PBS, incubated with the secondary antibodies diluted in blocking solution 1 hour at RT, washed two times with 0.1% Triton X-100/PBS and two times with PBS, and mounted on polysinated-coated slides with mounting medium Vectashield.

Images were obtained using a Leica TCS SL confocal microscope equipped with argon/He-Ne laser sources and an HCX PL APO CS 63.0×1.40 oil objective. During image acquisition, the 488 and 633 laser were set at 20% energy and the emission range was between 500–550 and 650 and 700 nm for Hyccin-Alexa 488 and NF-Alexa 633, respectively. The photomultiplier voltage gain was set to eliminate cell autofluorescence. Single plane images were taken at the center of cell thickness.

Cell immunofluorescence studies were performed in HeLa cells transiently transfected with a GFP-hyccin expression vector and in cultures of rat SC and DRG sensory neurons. Cells were fixed for 15 min in 4% paraformaldehyde in PBS at RT and then permeabilized with ice-cold methanol for 10 min at −20°C. Cells were incubated with 5% FBS in PBS-TRITON 0.3% for 30 min and then with the primary antibodies in a humidified box at 4°C ON. After three washes in PBS, cells were incubated with the secondary antibodies conjugated with Alexa Fluor 488 or 633 for 1 h. Following a PBS rinse, the coverslip (mounting with Vectashield reagent (Vector)) and the ACLAR dishes were observed at a confocal microscope.

Images were obtained using a Leica TCS SL confocal microscope equipped with argon/He-Ne laser sources and an HCX PL APO CS 63.0×1.40 oil objective. The photomultiplier voltage gain was set to eliminate cell autofluorescence. Single plane images were taken at the center of cell thickness.

Micrographs from DRG sensory neurons and primary SC were taken with an Olympus DP-70 digital microscope camera attached to an Olympus Provis AX-60 module fluorescence microscope with a 40× or 100× objective lens.

### Western blot

Lysates of HeLa cells, fibroblasts, brain, sciatic nerves and organotypic DRG cultures were prepared directly in ice cold RIPA buffer [50 mM Tris-HCl pH 7.4, 150 mM NaCl, 1% NP40, 0.25% Na-deoxycholate,1 mM PMSF, 1.100 proteinase inhibitor cocktail]. Samples were sonicated and recovered by centrifugation (5810 R; Eppendorf) for 15 min, at 12.000× g, 4°C. The total protein content was determined in supernatants by the Bio-Rad Protein Assay kit (Bio-Rad); equal protein amounts were loaded onto 12–15% polyacrilamide gel and separated by SDS-PAGE.

Proteins were transferred onto polyvinylidene difluoride membranes (Immobilon PVDF; Millipore). After the blocking reaction (5% BSA in PBS/0.1% Tween 20), membranes were incubated for 1 h at RT with primary antibody diluted in 1% BSA/PBS. Blots were rinsed and incubated with horseradish peroxidase rabbit anti-goat IgG secondary antibody and visualized using the ECL detection method (GE Health care). The sample protein signal was normalized to the respective GAPDH signal. For peptide competition assays, Hyccin (D-15) antibody diluted 1∶500 in 1% BSA/PBS was pre-incubated for 1 h at RT with 5 fold of the competing Hyccin D-15 homologous peptide and centrifuged (20,000× g for 15 min). The supernatant was applied onto a PVDF membrane to which the HeLa cells lysate had been transblotted.

### Electron Microscopy

Sciatic nerves were isolated from P2, P10, P30 and P60 Hyccin Het mice, fixed 1 h with 2% glutaraldehyde/PB, washed and incubated in Bluo-Gal staining solution (0.4 mg/ml Bluo-Gal, 3.1 mM potassium ferricyanide/ferrocyanide, 10 mM MgCl2, 0.12 M PB, pH 7.4) ON at 37°C After washing in PB, samples were post-fixed for 1 hr at RT with 1% osmium tetroxide in 0.06 M sodium cacodylate buffer. Nerves were dehydrated and rapidly infiltrated in propylene oxide and epoxy resin to minimize the solubilization of Bluo-Gal precipitates and then embedded. Ultrathin sections (70 nm) were performed using a Reichert ultramicrotome (Leica Microsystems, Wetzlar, Germany), collected on copper grids and not counterstained to better visualize Bluo-Gal crystals. All the samples were analysed under a JEM-1011 electron microscope (JEOL Ltd, Tokio, Japan) at 80 kV.

### Statistical analysis

Descriptive analyses were firstly performed; quantitative data were reported as median values with first and third quartiles (1^st^–3^rd^ q) and minimum and maximum value (Min-max). The comparison of quantitative data in different groups of observations was evaluated by the non parametric analysis of variance Kruskal-Wallis test); the *post-hoc* analysis was made by the Dunn's test.

All the statistical tests were two-sided and a p value less than 0.05 was considered as statistically significant. The software “Statistica” (StatSoft Co., Tulsa, OK, USA) was used for all the statistical analyses.

## Supporting Information

Figure S1 **Immunoblot analysis of hyccin.** Panel A: Total cellular lysates of fibroblasts from controls (C) and HCC affected individuals (Pt). Panel B: Total cellular lysates of Hela cells in the absence (−) or presence of competing peptide (+). An antibody against GAPDH was used as an internal control.
(TIF)

Methods S1 **Specific primers for quantitative real-time PCR of FAM126a (NM_001191969 rattus norvegicus, NM_053090 mus musculus), beta-actin (ACTB, NM_031144 rattus norvegicus, NM_007393 mus musculus), Glyceraldehyde-3-phosphate dehydrogenase (GAPDH, NM_017008 rattus norvegicus, NM_008084 mus musculus) and Myelin Protein Zero (MPZ, NM_017027).**
(DOC)
