# Supplementary figures and images for: Hyccin, the Molecule Mutated in the Leukodystrophy Hypomyelination and Congenital Cataract (HCC), Is a Neuronal Protein
Source: PLoS One. 2012 Mar 26;7(3):e32180. doi: 10.1371/journal.pone.0032180 (PMC3312879; doi:10.1371/journal.pone.0032180)

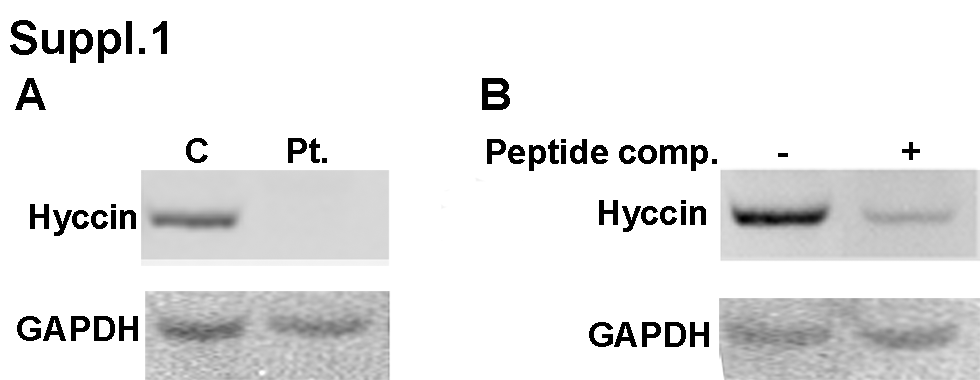

Supplement: Figure S1 — Immunoblot analysis of hyccin. Panel A: Total cellular lysates of fibroblasts from controls (C) and HCC affected individuals (Pt). Panel B: Total cellular lysates of Hela cells in the absence (−) or presence of competing peptide (+). An antibody against GAPDH was used as an internal control. (TIF) [file pone.0032180.s001.tif]
