# Supplementary material for: Hyccin, the Molecule Mutated in the Leukodystrophy Hypomyelination and Congenital Cataract (HCC), Is a Neuronal Protein
Source: PLoS One. 2012 Mar 26;7(3):e32180. doi: 10.1371/journal.pone.0032180 (PMC3312879; doi:10.1371/journal.pone.0032180)
Supplement: Methods S1 — Specific primers for quantitative real-time PCR of FAM126a (NM_001191969 rattus norvegicus, NM_053090 mus musculus), beta-actin (ACTB, NM_031144 rattus norvegicus, NM_007393 mus musculus), Glyceraldehyde-3-phosphate dehydrogenase (GAPDH, NM_017008 rattus norvegicus, NM_008084 mus musculus) and Myelin Protein Zero (MPZ, NM_017027). (DOC) [file pone.0032180.s002.doc]

**SUPPLEMENTARYMETHODS**

Methods S1

Specific primers for quantitative real-time PCR of FAM126a (NM_001191969 rattus norvegicus, NM_053090 mus musculus), β-actin (ACTB, NM_031144 rattus norvegicus, NM_007393 mus musculus ), Glyceraldehyde-3-phosphate dehydrogenase (GAPDH, NM_017008 rattus norvegicus, NM_008084 mus musculus ) and Myelin Protein Zero (MPZ, NM_017027).

| **Target gene** | **Accession number** | **Sequence** | **Product (bp)** |
| --- | --- | --- | --- |
| FAM 126a  rattus norvegicus | NM 001191969 | 5′-TGACAGCACAGAACAGGTTTGAAG-3′ | 195 |
|  |  | 5′-TGAAGCCAGATGAGATCGGTATCC-3′ |  |
| FAM 126a  mus musculus | NM_053090 | 5’-CACACCAACCTCCTCTAG-3’ | 120 |
|  |  | 5’-GTTATGTCCATCAGTTCTTCTT-5’ |  |
| β-actin  rattus norvegicus | NM_031144 | 5′- GGGAAATCGTGCGTGACATT-3′ | 76 |
|  |  | 5′- GCGGCAGTGGCCATCTC-3′ |  |
| β-actin  mus musculus | NM_007393 | 5’-GGCACCACACCTTCTACAATGAG-3’ | 161 |
|  |  | 5’-GACCAGAGGCATACAGGGACAG-3’ |  |
| GADPH  rattus norvegicus | NM_017008.3 | 5′-ATGACTCTACCCACGGCAAG-3′ | 89 |
|  |  | 5′-CTGGAAGATGGTGATGGGTT-3′ |  |
| GADPH  mus musculus | NM_008084 | 5’-TTGATGGCAACAATCTCCAC-3’ | 110 |
|  |  | 5’-CGTCCCGTAGACAAAATGGT-3’ |  |
| MPZ  rattus norvegicus | NM_017027 | 5′-CTGTTGCTGCTGTTGCTCTTCTAC-3′ | 182 |
|  |  | 5′-TTGGTGCTTCGGCTGTGGTC-3′ |  |
